# Endoscopic ultrasound-guided esophagojejunostomy of a complete anastomotic stricture

**DOI:** 10.1055/a-2387-4762

**Published:** 2024-09-04

**Authors:** Hui Lu, Tianyu Zhang, Ling Zhang, Dong Wang

**Affiliations:** 1 66281 Department of Gastroenterology, Shanghai Jiao Tong University Medical School Affiliated Ruijin Hospital, Shanghai, China

**Fig. 1:**
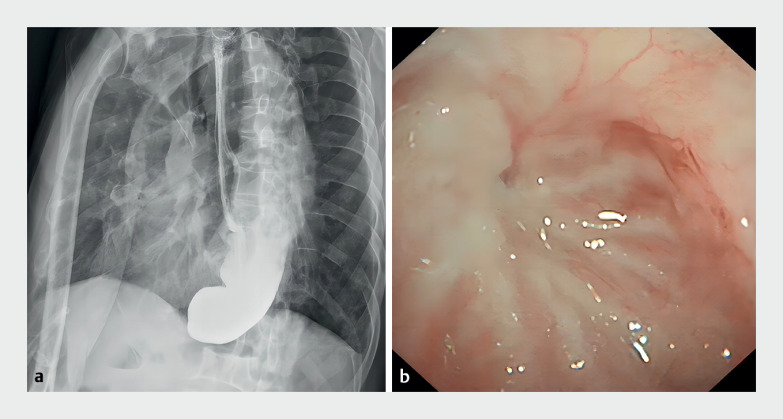
Complete esophagojejunal anastomotic stricture. **a** Esophagogram shows obvious dilation of the esophageal lumen and complete obliteration at the lower esophagus. **b** Gastroscopic view of scar tissue.

A 76-year-old man, diagnosed with anastomotic fistula after radical total gastrectomy and esophagojejunostomy (Roux-en-Y) due to gastric adenocarcinoma, was managed with thoracotomy and fistula repair. At 8 weeks after surgery, the patient was referred to our hospital because of progressive aphagia and persistent vomiting. Esophagography and gastroscopy revealed complete obstruction at the esophagojejunal anastomosis ( Fig. 1 ). The first attempt at endoscopic ultrasound (EUS)-guided rendezvous directly through the stricture was unsuccessful.

**Fig. 2:**
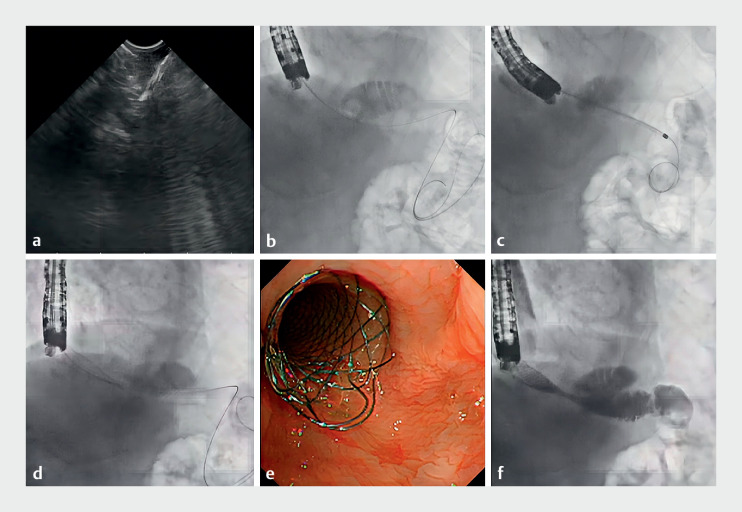
Recanalization to bypass the stricture using a biliary fully covered self-expanding metallic stent (FCSEMS) to create a new esophagojejunostomy. **a** A 19G needle was used to puncture the esophagus and enter the jejunal lumen. **b** A guidewire was passed into the efferent jejunal lumen under esophagography. **c** Bougies were used to dilate the passage. **d** A biliary FCSEMS was deployed along the guidewire through the passage. **e** Final gastroscopic view of the stent. **f** Contrast instilled into the stent flowed into the distal jejunal lumen without leakage.

Therefore, we attempted bypass recanalization to create a new esophagojejunostomy under EUS guidance ( Video 1 ). A forward-viewing echoendoscope was placed near the stricture and jejunal peristalsis was demonstrated on the EUS image. A 19G needle was used to puncture the esophageal wall and enter the jejunal lumen ( Fig. 2 **a** ). Contrast was instilled and fluoroscopy of the distal jejunum confirmed successful puncture. A guidewire was then passed through the needle into the efferent loop ( Fig. 2 **b** ). To avoid electrocautery risk to the thoracic aorta, a 6Fr and an 8.5Fr bougie were used separately to dilate a passage between the esophagus and jejunum ( Fig. 2 **c** ). Considering the diameter and maneuverability of the passage, we chose a biliary fully covered self-expanding metallic stent (FCSEMS, 10 × 80 mm) to deploy through the passage ( Fig. 2 **d, e** ). Instilled contrast was seen flowing into the distal jejunum without leakage ( Fig. 2 **f** ).

Endoscopic ultrasound-guided recanalization to bypass complete stricture of esophagojejunal anastomosis.Video 1

**Fig. 3:**
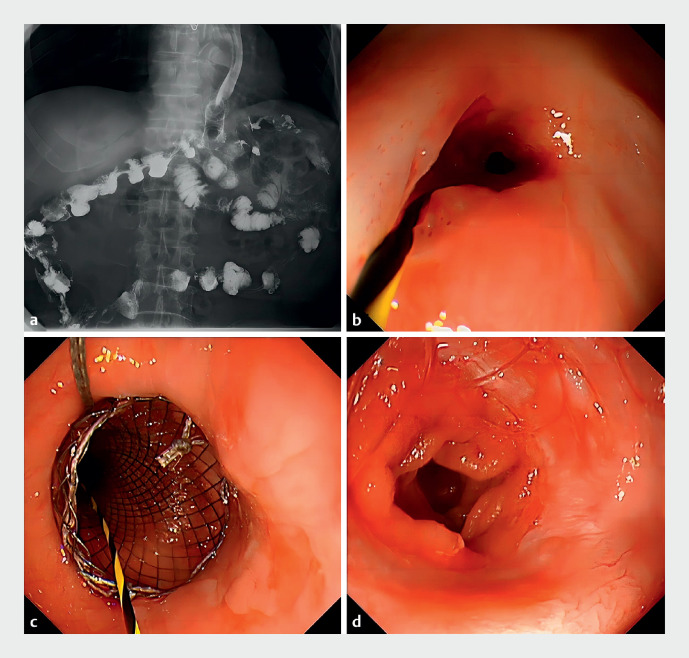
Stricture after EUS-guided bypass recanalization: **a–c** after 1 month, **d** after 4 months. **a** Fluoroscopy shows smooth flow through the new anastomosis. **b** Gastroscopic view of a clear and open passage after removal of the biliary FCSEMS. **c** Placement of the esophageal FCSEMS. **d** Ideal passage between the esophagus and jejunum after stent removal.

After 2 days, the patient was able to eat soft food without vomiting or pain. After 3 months, fluoroscopy showed smooth flow through the anastomosis, and the biliary FCSEMS was then replaced by an esophageal FCSEMS (20 × 80 mm). After 4 months, the esophageal stent was finally removed, leaving an ideal passage between esophagus and jejunum ( Fig. 3 ). No complications were seen during the follow-up.

To the best of our knowledge, this is the first case report of EUS-guided recanalization bypassing the stricture of a complete esophageal stenosis. It may be a promising recanalization method to treat esophageal stenosis when conventional approaches fail.

Endoscopy_UCTN_Code_TTT_1AQ_2AF

